# Evaluative and emotional routes to audience intention in musical-theater singing: An integrated response architecture

**DOI:** 10.3389/fpsyg.2026.1870336

**Published:** 2026-08-04

**Authors:** Lujing Zheng, Hao Chen, Yaqiong Guo

**Affiliations:** 1 City University Malaysia, Faculty of Creative Design, Petaling Jaya, Malaysia; 2 Zhengzhou Shengda University, College of Art, Zhengzhou, Henan, China

**Keywords:** audience response, emotional perception, music psychology, musical theater, performance psychology, vocal appraisal

## Abstract

Audience responses to vocal performances involve complex psychological processes beyond general aesthetic liking. However, less is known about how vocal appraisal, perceived emotional meaning, and future engagement intention are related. This study examined how technical-expressive perception (TEP), emotional perception (EP), and audience intention (AI) are related in responses to musical-theater singing. We used a single-wave survey of 306 adult musical-theater listeners in China to test their associations and compare them across attendance-frequency groups. Technical-expressive perception was strongly associated with emotional perception. Emotional perception was more closely related to audience intention than technical-expressive perception. In the indirect-association model, the remaining link between technical-expressive perception and audience intention was no longer reliable after emotional perception was included. This suggests that emotional perception was more closely tied to future engagement intention. Attendance frequency was related to technical-expressive perception and emotional perception, but not to audience intention. More frequent attendees reported higher vocal appraisal and emotional perception, whereas audience intention did not differ reliably across groups. These findings show that technical-expressive perception, emotional perception, and audience intention were related but not equivalent. The study’s main contribution is the Audience Response Architecture, which explains how these three responses are connected but play different roles in responses to musical-theater singing. This framework contributes to music and performance psychology by showing why studies of audience intention should distinguish between how listeners appraise vocal delivery and how they perceive emotional meaning.

## 1 Introduction

Musical-theater singing provides a rich context for examining the perceptual and affective processes underlying audience responses to performed voice. In this genre, vocal performance is perceived not merely as technical display, but as a way to communicate character, emotion, and dramatic situation. Listeners may therefore appraise vocal delivery and judge whether it communicates emotional meaning clearly. These responses may then be related to their future engagement intention. This question is also practically relevant because musical theater has become an increasingly visible segment of the live-performance sector. In China, for example, the musical-theater market staged nearly 19,700 performances in 2025, attracting 8.19 million audience visits and generating about 1.8 billion yuan in box-office revenue. Similar signs of sustained demand have also been observed in South Korea and Australia. However, market expansion alone cannot explain audience engagement. Attendance-related responses also depend on how audiences evaluate the performance experience and the emotional value it provides (Kawase and Obata, 2016; Ulziibadrakh et al., 2025).

In performance contexts, audience engagement depends not only on production supply or genre popularity, but also on how artistic experience is received and valued by audiences (Gaztelumendi and Huaman-Ramirez, 2024). This issue is especially relevant to musical-theater singing because sung performance communicates through voice, text, emotion, and stage situation. These elements are heard together rather than separately (Morgan, 2025). Audiences may therefore respond to musical-theater singing in several ways. They may evaluate the quality of vocal delivery, perceive whether emotional meaning is communicated clearly, and form a future-oriented intention to attend again or support similar performances (Greenspon and Montanaro, 2023; Li and Hu, 2025; Moura et al., 2024). This makes musical-theater singing a suitable context for examining how evaluation, perceived emotion, and later intention may be organized within audience experience.

Future-oriented audience outcomes matter in live performance because they show whether an experience is linked to later willingness to revisit or pay for similar events. Such responses have also been linked to how audiences perceive and value performance experiences (Sengoz et al., 2024). More broadly, recent work on concert and arts audiences suggests that audience experience cannot be reduced to a single evaluative judgement, but varies across different experiential dimensions and performance formats (Wald-Fuhrmann et al., 2026). However, in musical-theater singing, these response dimensions have rarely been examined together. Existing work more often addresses performance evaluation, vocal emotion perception, or sung-text intelligibility as separate topics (Kondo et al., 2025; Nussbaum et al., 2024; Vurma et al., 2023). Less attention has been paid to how evaluation, perceived emotion, and future-oriented audience intention are related within audience response. The present study addresses this gap in the context of musical-theater singing.

Against this background, the present study examines audience response to musical-theater singing by focusing on technical-expressive perception (TEP), emotional perception (EP), and audience intention (AI). Using an online survey of adult musical-theater listeners, it examines how audiences rate vocal delivery, perceive emotional meaning, and express willingness to return. It also tests whether these responses differ across attendance-frequency groups.

In practical terms, the study helps clarify how listeners perceive musical-theater singing. Rather than treating vocal delivery as an isolated display of technical skill, the study considers how audiences connect vocal delivery with expressive clarity and emotional communication. In this sense, the study is relevant to audience research and to performance practice concerned with how sung delivery is received by listeners.

Theoretically, this study proposes an Audience Response Architecture for musical-theater singing. It treats technical-expressive perception, emotional perception, and audience intention as related responses that play different roles. It also contributes to emotions research by examining how listeners in a Chinese musical-theater context connect vocal evaluation, perceived emotion, and future intention. These aims lead to the following research questions: RQ1: How are technical-expressive perception (TEP), emotional perception (EP), and audience intention (AI) associated within audience responses to musical-theater singing? RQ2: Do listeners with different attendance frequencies differ in their levels of TEP, EP, and AI?

## 2 Theoretical background

### 2.1 Core constructs

In the broader literature, judgments of sung performance are usually discussed as performance assessment, overall vocal evaluation, or vocal quality judgment. They are not usually treated as a single established construct equivalent to technical-expressive perception. Research on music-performance assessment shows that evaluation systems are typically multidimensional and are commonly organized around technical and interpretative or expressive criteria rather than around one isolated standard (Moura et al., 2024). Related work on singing evaluation further indicates that overall judgments may depend on several vocal and perceptual features, including those associated with vocal control and expressiveness, rather than on technical accuracy alone (Kondo et al., 2025). In addition, research on sung-text intelligibility shows that the communicative effectiveness of singing also depends in part on whether verbal content can be clearly perceived by listeners (Vurma et al., 2023). However, no single established term in the current literature fully captures the combination of technical control, expressive adequacy, textual clarity, and stylistic fit required in musical-theater singing. For this reason, the present study uses the term technical-expressive perception (TEP). TEP refers to listeners’ judgment of whether vocal delivery sounds technically controlled. It also captures whether the delivery works as expressive and communicative performance in a musical-theater context.

In the literature on singing and music perception, listeners’ responses to emotion are usually discussed through vocal emotion recognition, perceived emotional expression, and judgments of emotional genuineness. They are less often treated as a single construct labeled emotional perception. Research on vocal emotion recognition shows that listeners can identify emotional information in the singing voice and that this ability is tied to sensitivity to vocal cues (Greenspon and Montanaro, 2023). Related work on perceived emotional expression further indicates that emotional communication in music depends on combinations of cues rather than on any single acoustic feature in isolation, with cue patterns shaping how emotion is heard by listeners (Micallef Grimaud and Eerola, 2022; Nussbaum et al., 2024). In musical-theater performance, the issue becomes more specific still: listeners may judge not only whether an emotion is expressed, but whether that expression sounds genuine and fits the character’s situation within acted song (Morgan, 2025). For this reason, the present study uses the term emotional perception (EP). The term is used because the study focuses on how listeners receive emotional meaning, not only on whether they identify a basic emotion or infer performer intention. Accordingly, EP refers to the extent to which emotional meaning in musical-theater singing is received by audiences as clear, salient, and believable.

Repurchase intention (RI) is commonly defined as a consumer’s willingness to purchase the same product or service again after prior consumption experience. The concept has been widely used in research on post-consumption behavior and customer loyalty (Hellier et al., 2003). More broadly, RI belongs to the tradition of behavioral intention research, in which intention is treated as the most immediate psychological antecedent of later behavior rather than as behavior itself (Ajzen, 1991; Bosnjak et al., 2020). Existing work has also shown that post-consumption evaluations and emotional responses, including satisfaction, can shape later behavioral intentions such as patronage and willingness to purchase (Hellier et al., 2003; Ladhari et al., 2017). However, RI in its narrower consumer sense is not fully adequate for the purposes of the present study. The focus here is not limited to whether audiences would repurchase the same theatrical product in a strict transactional sense. Instead, the study asks whether current response to musical-theater singing is linked to later willingness to attend again or support similar performances. For this reason, the broader term audience intention (AI) is used. AI refers to a future-oriented willingness to re-engage with similar live musical-theater events, particularly through return attendance or support for similar performances. It is therefore treated as a behavioral intention outcome for audiences. It is distinct from immediate evaluation and emotional perception, although it may be related to both.

### 2.2 Communication framework

A communication-based perspective provides a useful lens for understanding audience response to musical-theater singing. In research on music and vocal communication, emotion is understood not only as a listener response, but also as something conveyed through audible cues and recognized by listeners (Greenspon and Montanaro, 2023; Juslin, 2013; Juslin and Laukka, 2003). More recent work on vocal emotion perception further suggests that such communication depends on combinations of acoustic cues, especially fundamental frequency and timbre, rather than on any single feature in isolation (Nussbaum et al., 2024). This perspective is especially relevant in musical theater, where singing involves sound quality, style, expression, and acted performance. Listeners may judge whether the emotion in a sung role sounds genuine and fits the character’s situation (Morgan, 2025). In the present study, this framework guides the examination of audience response to musical-theater singing. It helps explain how different audience responses components may be related in musical-theater listening without assuming a fixed causal model.

Within such a framework, audience response to musical-theater singing involves more than one listener process. Research on music communication has shown that performance does not simply transmit sound, but can generate a performer–audience shared experience in which expressive intent is perceived and interpreted by listeners (James, 2018). Empirical work on musical performance likewise indicates that audience response may include both affective and cognitive dimensions rather than one undifferentiated judgment (Broughton et al., 2021). Studies of perceived emotion in musical performance further suggest that listeners’ judgments are shaped by combinations of auditory and movement-related features. Emotional response is therefore linked to how expressive information is conveyed in performance (Thompson et al., 2023). From this perspective, technical-expressive perception and emotional perception can be treated as related but distinguishable audience responses: one concerns how performance delivery is judged, whereas the other concerns how emotional meaning is received.

### 2.3 Audience response structure

Music-theater audiences do not respond to singing in only one way. Research on music-performance assessment suggests that judgments of performance quality are rarely based on a single criterion. Performance-assessment systems are typically organized around both technical and expressive dimensions, which means that listener evaluation extends beyond technical correctness alone (Moura et al., 2024). A similar pattern has been reported in singing evaluation, where overall judgments in opera singing depend on multiple vocal characteristics rather than on one isolated feature (Kondo et al., 2025). These studies suggest that when audiences evaluate singing, they respond not only to accuracy, but also to how vocal delivery functions as performance.

A second line of research has examined how listeners perceive emotional meaning in music and voice. Emotional expression is not conveyed by one cue in isolation; rather, it emerges through interactions among expressive cues (Micallef Grimaud and Eerola, 2022). In vocal contexts, emotional perception appears to depend especially on combinations of fundamental frequency and timbre (Nussbaum et al., 2024). From this perspective, emotional meaning is heard through combinations of expressive cues in the voice, not through any single feature alone.

This issue becomes especially important in musical-theater singing. In acted song, listeners may judge not only whether emotion is present, but also whether it sounds genuine and fits the character’s situation (Morgan, 2025). Audience response therefore seems to involve two closely connected but distinguishable aspects: how singing is delivered and what emotional meaning that delivery communicates.

A further question concerns what may follow from this experience. Studies of audience engagement indicate that musical performance involves both affective and cognitive responses (Broughton et al., 2021). In addition, perceived emotions in musical performance have been linked to combined audio and movement features (Thompson et al., 2023). Beyond immediate perception, post-experience evaluations can shape continuance intention (Zhao et al., 2012). Emotional satisfaction can influence behavioral intentions such as patronage and purchase-related willingness (Ladhari et al., 2017). Although these studies were not conducted specifically in musical-theater singing, they support the broader point that later audience intention is related to evaluative and emotional response.

This leaves a clear gap in the literature. Performance evaluation, emotional perception, and later-oriented intention have all been studied, but they have more often been examined separately than as parts of the same audience-response structure. For this reason, the present study examines technical-expressive perception (TEP), emotional perception (EP), and audience intention (AI) together. In this structure, TEP concerns how vocal delivery is appraised, EP concerns how emotional meaning is received, and AI concerns whether that experience extends into a later willingness to re-engage with similar performances. In the present study, this overall arrangement is described as an Audience Response Architecture. The term is not used to imply a fixed causal model. It is used to indicate that these three responses are related, but do not play the same role in audience response.

### 2.4 Attendance frequency

Attendance frequency also warrants attention as a source of audience variation. Research on arts audiences suggests that regular attenders do not approach performances in the same way as first-time or occasional attenders. Repeated participation is associated with different expectations, frames of reference, and forms of engagement with live events (Pitts, 2016). In music listening more broadly, familiarity and repeated exposure have been shown to shape liking and related evaluative judgments, suggesting that prior exposure can influence how listeners respond to what they hear (Madison and Schiölde, 2017; Smit et al., 2022). These findings do not imply that more frequent attenders necessarily experience different emotions in any simple or uniform way. They do suggest that repeated exposure may influence what audiences notice, value, and evaluate in performance. In the present study, attendance frequency is treated as a way to describe audience variation. It is included to examine whether higher- and lower-frequency attendees differ in technical-expressive perception (TEP), emotional perception (EP), and audience intention (AI). Particular attention to TEP as a response component that may be especially sensitive to differences in prior attendance.

## 3 Methods

### 3.1 Design overview

This study employed an online questionnaire survey to examine audience responses to musical-theater singing. The questionnaire measured three listener-side components: technical-expressive perception (TEP), emotional perception (EP), and audience intention (AI). It also collected demographic and attendance-frequency information for group comparisons. The analysis first evaluated the measurement quality of the retained items. It then examined the relations among the three response components and tested whether response levels differed across attendance-frequency groups. Because all variables were collected in one survey wave, the findings are interpreted as association and group differences rather than temporal or causal effects.

### 3.2 Participants

An online questionnaire was hosted on the Wenjuanxing survey platform and distributed through musical-theater-related social-media accounts, university mailing lists, and fan communities. Data were collected between 7 December 2024 and 8 May 2025. Participants were recruited using convenience sampling with purposive targeting of individuals who reported an interest in musical theater or related performing arts. Eligibility criteria were being aged 18 years or above, reading the information sheet, providing electronic consent, and completing all required items. No incentives were offered for participation. This recruitment strategy was intended to reach respondents with enough familiarity or interest to evaluate musical-theater singing in a survey setting.

Data-quality checks were applied prior to analysis. Responses with completion times below 120 s (*n* = 8) were excluded as likely insufficient for attentive responding. Likely duplicate submissions identified through browser cookies and IP addresses were removed (*n* = 2). This process yielded 309 valid responses. Although the survey included an adult eligibility screen, three respondents later self-reported as being under 18. These cases were removed before inferential analysis, resulting in a final analytic sample of 306 adult respondents. Because all main questionnaire items had to be completed before submission, there were no missing data for the main survey variables. Given the convenience and interest-based recruitment strategy, the sample should be viewed as a non-probability sample of engaged musical-theater listeners. It should not be treated as a representative cross-section of the general population.

For inferential age-group comparisons, analyses focused on the 18–30 and 31–45 groups. The ≥46 group was retained for descriptive reporting but excluded from inferential group comparisons because of its small cell size. To support stable estimation in group comparisons, musical-theater attendance frequency was collapsed into three ordered levels: <1, 1–3, and ≥4 times/year. Higher values indicated more frequent attendance.

### 3.3 Measures

The questionnaire was administered in Mandarin Chinese. No established scale directly captured the present constructs in the context of musical-theater listening. The items were developed for this study with reference to prior literature on vocal performance evaluation, perceived emotional meaning in voice and music, and audience response and future attendance intention (Fine and Ginsborg, 2014; Juslin and Laukka, 2003; Kawase and Obata, 2016; Moura et al., 2024; Quinto et al., 2013). Item wording was designed to reflect technical-expressive perception (TEP), emotional perception (EP), and audience intention (AI) while remaining understandable to non-specialist respondents. No separate pilot sample was collected. Instead, three reviewers with academic or professional experience in music, voice, or musical-theater research and practice examined the draft items for wording, clarity, and representativeness of the intended constructs. Minor revisions were then made on the basis of their feedback to improve readability and contextual fit without changing the core construct meaning.

The questionnaire consisted of one background section and three substantive sections. Section A included demographic and exposure variables used for sample description and group comparisons. Section B comprised three TEP items (Q5–Q7), Section C comprised four emotion-related items (Q8–Q11), and Section D comprised four future-engagement-related items (Q12–Q15). For analysis, EP was indexed by Q8–Q10, and AI by Q12 and Q15. Q11, Q13, and Q14 were treated as descriptive items and were not included in composite scores (see Table 1).

**TABLE 1:** Overview of questionnaire constructs and analytic item sets.

Construct	Total items	Core items	Construct focus	Literature basis
TEP	3	Q5–Q7	Technical control, expressive support, clarity, stylistic appropriateness	Moura et al., 2024; Huang et al., 2024; Morgan, 2025
EP	4	Q8–Q10	Emotional clarity, emotional genuineness, perceived emotional meaning	Greenspon and Montanaro, 2023; Morgan, 2025; Nussbaum et al., 2024
AI	4	Q12, Q15	Future engagement intention, including return attendance and willingness to support similar performances	Li and Hu, 2025; Conner and Norman, 2022

Total items refer to the number of questionnaire items originally included in each section. Core items refer to the subset retained for composite analysis. Q11, Q13, and Q14 were analyzed descriptively and were not included in composite scores.

Responses were recorded using item-specific 5-point response anchors. Because the original questionnaire presented options from stronger to weaker endorsement, exported responses were reverse-coded prior to analysis using x′ = 6 – x. After reverse-coding, higher analyzed scores consistently indicated stronger endorsement across all eight core items. Composite scores were then computed as the mean of the reverse-coded items (TEP = mean of Q5–Q7; EP = mean of Q8–Q10; AI = mean of Q12 and Q15). Non-scale questions were analyzed descriptively and were not combined into indices. The full English translation of the questionnaire is provided in the Supplementary material.

### 3.4 Data analysis

All survey analyses were conducted in SPSS Statistics, and the indirect-association models were estimated using Hayes’ PROCESS macro. Factor structure was examined using principal-axis factoring with Promax rotation. The questionnaire was designed around three focal constructs, a three-factor solution was specified *a priori*. This solution was evaluated using the KMO index, Bartlett’s test of sphericity, extracted communalities, and the rotated loading pattern. Internal consistency was assessed using Cronbach’s α for TEP and EP and the Spearman–Brown coefficient for AI. As a supplementary measurement check, confirmatory factor analysis (CFA) compared one-factor, two-factor, and three-factor models. Convergent validity was assessed using composite reliability (CR) and average variance extracted (AVE).

Descriptive statistics and Pearson correlations were used to summarize associations among TEP, EP, and AI. Group differences by gender, age, educational background, and attendance frequency were examined using independent-samples t-tests or one-way analysis of variance (ANOVA), as appropriate. When Levene’s test indicated unequal variances, Welch’s test was used, followed by Games–Howell post-hoc comparisons where pairwise comparisons were required. To reduce the risk of inflated Type I error in multiple group comparisons, *p* values were adjusted using the Benjamini–Hochberg procedure with *q* = 0.05. All tests were two-tailed, with statistical significance evaluated at α = 0.05 unless otherwise noted.

To examine whether the observed association was consistent with the proposed Audience Response Architecture, an indirect-association model was estimated using PROCESS Model 4 with 5,000 bootstrap resamples. In this model, EP was specified as the intervening variable linking TEP and AI. No demographic covariates were entered in this model. The aim was to describe the basic association structure among the three focal constructs rather than to estimate adjusted population effects. Because all variables were measured in the same survey wave, this model was interpreted as evidence of associations rather than causal process. As a brief robustness check on the emotional-perception operationalization, the model was re-estimated using the outcome-focused item EP2 as the intervening variable. This item asked whether emotional change could be perceived through the actor’s singing.

Attendance frequency was examined in two ways. It was used as a grouping variable in mean-comparison analyses across low-, medium-, and high-attendance groups. It was also tested as an exploratory moderator in a PROCESS Model 7 analysis of the TEP–EP association. Variables involved in the interaction term were mean-centered before analysis. Given the cross-sectional design, the interaction model was interpreted cautiously as exploratory evidence that the TEP–EP association varied by attendance frequency.

## 4 Results

### 4.1 Sample

After data screening, the final analytic sample comprised 306 respondents. The sample was predominantly young, female, and highly educated: 73.9% were aged 18–30 years, 68.3% were female, and 97.7% held a bachelor’s degree or above. In terms of attendance frequency, the largest group reported attending musical theater 1–3 times per year (52.3%), followed by the low-attendance group (<1 time/year, 34.0%) and the high-attendance group (≥4 times/year, 13.7%). Overall, this profile indicates that the sample was concentrated among younger and relatively highly educated respondents rather than evenly distributed across audience segments. This uneven distribution was considered when interpreting demographic group comparisons. For inferential analyses, age-group comparisons focused on the 18–30 and 31–45 groups. Respondents aged 46 years and above were retained for descriptive reporting only because of the small cell size (*n* = 13). Educational comparisons focused on the bachelor and master’s-and-above groups. The high-school-or-below group was excluded from inferential comparisons because of its small size (*n* = 7). Detailed demographic characteristics of the final analytic sample are presented in Table 2.

**TABLE 2:** Demographic characteristics of the respondents (*n* = 306).

Variable	Category	Frequency (*n*)	Percentage (*%*)
Gender	Male	97	31.7%
	Female	209	68.3%
Age	18–30 years	226	73.9%
	31–45 years	67	21.9%
	46 years and above	13	4.2%
Educational background	High school or below	7	2.3%
	Bachelor	252	82.4%
	Master and above	47	15.4%
Musical-theater attendance frequency	Low (<1 time/year)	104	34.0%
	Medium (1–3 times/year)	160	52.3%
	High (≥4 times/year)	42	13.7%

Percentages were calculated from the final analytic sample (*n* = 306). Musical-theater attendance frequency refers to the self-reported number of musical-theater performances attended per year.

### 4.2 Measurement quality

Factorability and internal consistency were first examined to assess whether the study-specific items reflected the intended three-construct structure. Principal-axis factoring with Promax rotation indicated that the eight core items were suitable for factor analysis (KMO = 0.856; Bartlett’s χ^2^(28) = 882.77, *p* < 0.001). Because the questionnaire was designed around three focal constructs, a three-factor solution was specified *a priori*. It was evaluated for interpretability rather than identified solely on an unrestricted eigenvalue criterion. This solution matched the three intended constructs: technical-expressive perception (TEP), emotional perception (EP), and audience intention (AI). It accounted for 56.00% of the extracted variance. Extracted communalities ranged from 0.492 to 0.609. Primary pattern-matrix loadings ranged from 0.411 to 0.855. Most items loaded most strongly on their intended factor, although one emotional-perception item showed a modest cross-loading. Overall, the rotated solution supported the intended three-construct interpretation. Internal consistency was acceptable for all three constructs (TEP α = 0.781; EP α = 0.740; AI Spearman–Brown ρ = 0.744). Factorability and reliability diagnostics are reported in Table 3.

**TABLE 3:** Factorability and reliability diagnostics for the survey items (*n* = 306).

Diagnostic	Value	Common guideline
Cronbach’s α (TEP)	0.781	≥0.70
Cronbach’s α (EP)	0.740	≥0.70
Spearman–Brown (AI)	0.744	≥0.70
KMO (overall)	0.856	≥0.60
Bartlett’s test	χ^2^(28) = 882.766, *p* < 0.001	*p* < 0.05
Variance explained (3-factor solution)	56.00%	>50% preferred
Communalities (range)	0.492–0.609	≥0.20

Principal-axis factoring with Promax rotation (Kaiser normalization) was used. A three-factor solution was specified *a priori* in line with the intended TEP–EP–AI structure. Variance explained refers to the cumulative percentage under Extraction Sums of Squared Loadings. Communalities refer to the extracted common variance for each item. Cronbach’s α is reported for TEP and EP, whereas the Spearman–Brown coefficient is reported for the two-item AI scale.

As a supplementary measurement check, confirmatory factor analysis (CFA) was used to compare one-factor, two-factor, and theory-specified three-factor models for the eight core items. The three-factor model showed the best overall fit, outperforming both the one-factor and two-factor alternatives. Although the constructs were substantially interrelated, the model comparison supported treating the three constructs as related but distinguishable. The results did not support reducing them to one general response factor or to a two-factor structure. Table 4 summarizes the model-comparison results.

**TABLE 4:** Confirmatory factor analysis model comparison for the eight core items (*n* = 306).

Model	*χ* ^2^	*df*	CFI	TLI	RMSEA	Interpretation
One-factor model	156.37	20	0.843	0.780	0.150	Poor fit
Two-factor model	70.09	19	0.941	0.913	0.094	Improved but still weaker
Three-factor model (TEP–EP–AI)	39.92	17	0.974	0.956	0.066	Best fit

The one-factor model specified a single general audience-response factor. The two-factor model combined Q5–Q10 into a single performance/emotion factor and retained AI as a second factor. The three-factor model specified separate but correlated TEP, EP, and AI factors. Better fit is indicated by lower χ^2^ and RMSEA and higher CFI/TLI.

Convergent validity was further assessed using composite reliability (CR) and average variance extracted (AVE). All three constructs met the conventional threshold for composite reliability (CR > 0.70) (see Table 5). AVE values exceeded.50 for TEP and AI. The EP value was slightly below this threshold but close to it, suggesting acceptable convergence when considered alongside the overall model pattern. Given the brief item set and the conceptual proximity between technical-expressive perception and emotional perception, these results are interpreted as supportive rather than definitive evidence of construct-level separation.

**TABLE 5:** Standardized loadings and convergent-validity indices for the retained three-factor model.

Construct	Items	Standardized loadings	CR	AVE
TEP	Q5_R, Q6_R, Q7_R	0.727, 0.779, 0.718	0.784	0.548
EP	Q8_R, Q9_R, Q10_R	0.735, 0.708, 0.658	0.743	0.492
AI	Q12_R, Q15_R	0.787, 0.752	0.755	0.607

CR, composite reliability; AVE, average variance extracted. CR values above.70 were treated as acceptable, and AVE values above 0.50 as indicative of adequate convergent validity. EP fell slightly below the AVE guideline, so its convergent validity should be interpreted cautiously in light of the brief item set and the overall model pattern.

### 4.3 Associations

To describe the basic association among the three focal constructs, zero-order correlations were first examined. Technical-expressive perception (TEP) was strongly associated with emotional perception (EP) (*r* = 0.660, *p* < 0.001) and modestly associated with audience intention (AI) (*r* = 0.325, *p* < 0.001). Emotional perception was moderately associated with audience intention (*r* = 0.486, *p* < 0.001). Overall, the correlation pattern showed that EP was more closely related to AI than TEP was. Full correlation results are presented in Table 6.

**TABLE 6:** Correlations among TEP, EP, and AI (*n* = 306).

Variable	1	2	3
1. Technical–Expressive Perception (TEP)	1	0.660**	0.325**
2. Emotional Perception (EP)	0.660**	1	0.486**
3. Audience Intention (AI)	0.325**	0.486**	1

Values are Pearson correlation coefficients. All tests were two-tailed. All non-diagonal correlations were significant at *p* < 0.001.

An indirect-association model was then estimated using PROCESS Model 4 with 5,000 bootstrap samples. This model examined whether the observed TEP–AI association was consistent with an intervening role for emotional perception. No demographic covariates were entered in this model. The aim was to describe the basic association structure among the three focal constructs rather than to estimate adjusted population effects. TEP was positively associated with EP (*B* = 0.633, *SE* = 0.041, *p* < 0.001), and EP was positively associated with AI after adjustment for TEP (*B* = 0.669, *SE* = 0.093, *p* < 0.001). The total association between TEP and AI was also significant (*B* = 0.434, *SE* = 0.072, *p* < 0.001). The remaining direct association between TEP and AI was not reliable (*B* = 0.011, *SE* = 0.089, *p* = 0.900). The indirect component through EP was significant (*B* = 0.423, Boot *SE* = 0.073, 95% CI [0.292, 0.573]). This result was consistent with an indirect link through emotional perception. As a robustness check, the model was re-estimated using EP2 as the intervening variable. EP2 was the outcome-focused emotional-perception item concerning whether emotional change could be perceived through the actor’s singing. The bootstrap confidence interval for the indirect component again excluded zero. In this item-level specification, however, the remaining direct association between TEP and AI was larger and remained statistically reliable (see Table 7).

**TABLE 7:** Robustness check using EP2 as the intervening variable.

Effect type	Path / quantity	*B*	*SE*	*p*	95% CI
a path	TEP_m → EP2	0.651	0.0543	<0.001	[0.5445, 0.7583]
b path	EP2 → AI_m (controlling TEP_m)	0.220	0.0755	0.0038	[0.0715, 0.3686]
Total effect (c)	TEP_m → AI_m	0.434	0.0724	<0.001	[0.2920, 0.5769]
Direct effect (c′)	TEP_m → AI_m (controlling EP2)	0.291	0.0868	0.0009	[0.1203, 0.4619]
Indirect effect (ab)	TEP_m → EP2 → AI_m	0.143	0.0592	–	[0.0286, 0.2616]

PROCESS Model 4 with 5,000 bootstrap samples; *n* = 306. EP2 refers to the outcome-focused EP item (Q9, reverse-coded). For the indirect effect, SE is the bootstrap standard error and the 95% CI is the bootstrap confidence interval.

### 4.4 Group differences

**FIGURE 1:**
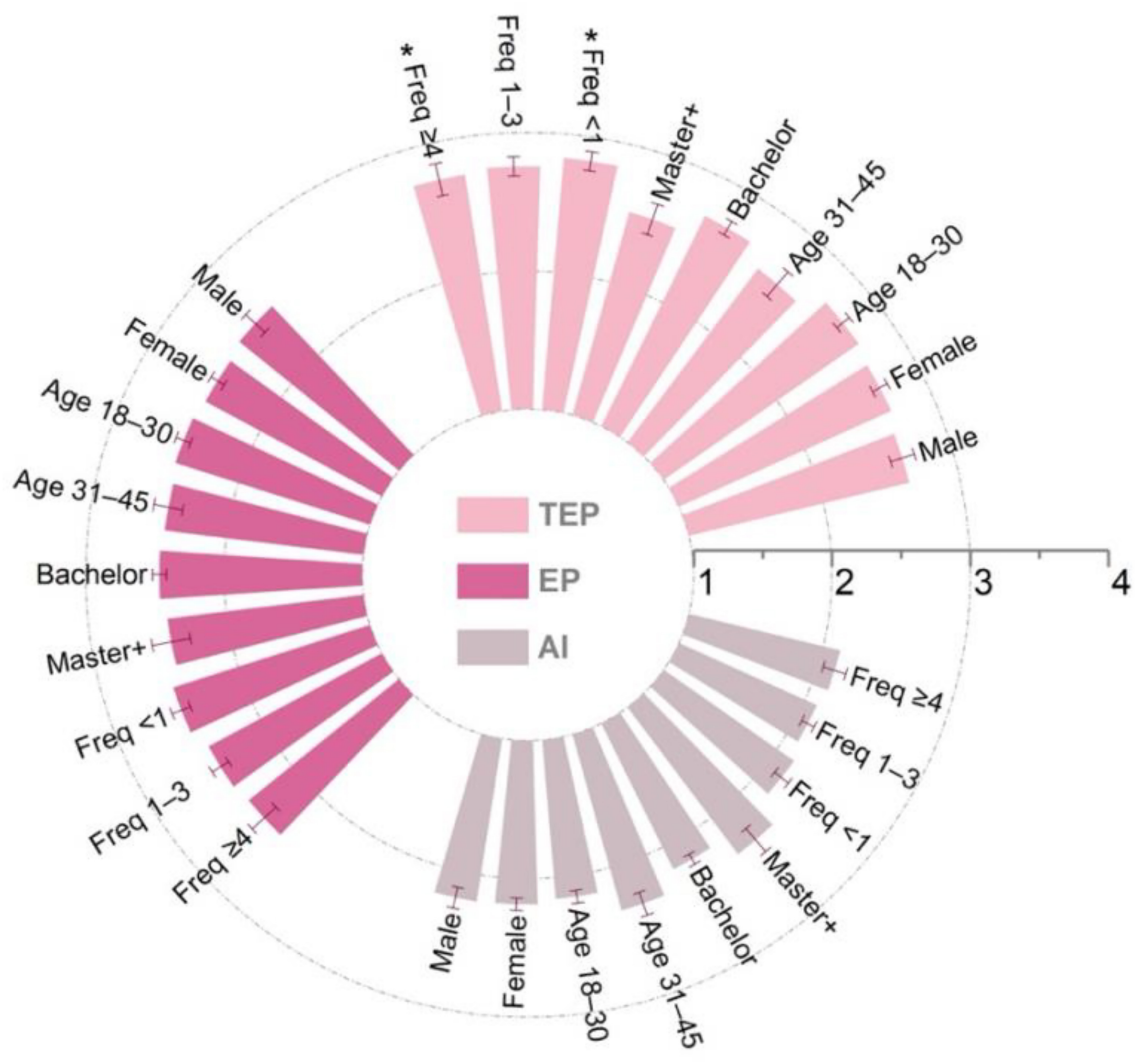
Mean scores for TEP, EP, and AI across audience groups (*n* = 306). Means are shown on 5-point scales. Error bars represent standard deviations. Asterisks denote pairwise contrasts that remained significant after Benjamini–Hochberg false-discovery-rate correction (*q* = 0.05). Welch corrections were applied where variances were unequal.

Group comparisons were conducted across gender, age, educational background, and attendance frequency to examine whether attendance frequency showed a distinct pattern in the focal audience responses. Because group sizes were unequal across several demographic categories, Welch’s test and Games–Howell *post-hoc* comparisons were used whenever homogeneity of variance was not met. In this sample, no reliable group differences were detected by gender, age, or educational background (all *p* ≥ 0.13; small effects). Given the uneven subgroup sizes and restricted demographic range, these non-significant findings should be interpreted cautiously. By contrast, attendance frequency showed significant mean differences in the focal audience-response measures. Specifically, attendance frequency was associated with small but significant differences in technical-expressive perception (TEP) and emotional perception (EP). For technical-expressive perception, Welch’s *F*(2, 115.49) = 3.93, *p* = 0.022, *η*^2^ = 0.026. For emotional perception, Welch’s *F*(2, 115.62) = 4.27, *p* = 0.016, *η*^2^ = 0.025. Audience intention (AI) did not differ significantly across attendance groups (*F*(2, 303) = 1.93, *p* = 0.147, *η*^2^ = 0.013). Descriptively, mean scores increased across attendance groups for all three constructs, with the highest values observed in the ≥4/year group and the lowest in the <1/year group. After Benjamini–Hochberg correction, Games-Howell comparisons showed that respondents in the ≥4/year group reported significantly higher TEP (*M* = 4.59) and EP (*M* = 4.48) than those in the <1/year group (TEP: *M* = 4.32; EP: *M* = 4.21). No other pairwise contrasts were significant. Group means with standard-deviation error bars are shown in Figure 1.

The primary attendance-related analysis focused on between-group differences in mean response levels. As a supplementary exploratory step, an interaction model examined whether the strength of the TEP–EP association varied by attendance frequency. The TEP × attendance interaction predicting EP was small but statistically significant (*B* = −0.127, *SE* = 0.064, *p* = 0.047; Δ*R*^2^ = 0.007). Simple-slope estimates indicated that the positive association between TEP and EP became weaker as attendance frequency increased. The estimated effects of 0.684, 0.600, and 0.516 at low (−1 SD), mean, and high (+1 SD) levels of attendance frequency, respectively. Given the small effect size, however, this pattern should be treated cautiously as exploratory evidence rather than as a central attendance-related finding.

## 5 Discussion

### 5.1 Main findings

The discussion examines the relations among technical-expressive perception (TEP), emotional perception (EP), and audience intention (AI), together with attendance-related variation in these responses. The first group of findings showed that the three responses were related but did not play the same role. Technical-expressive perception was strongly related to emotional perception. Emotional perception was more closely related to audience intention than technical-expressive perception was. The indirect-association model showed the same pattern. Once emotional perception was taken into account, the remaining link between technical-expressive perception and audience intention was not reliable. In this sample, gender, age, and educational background did not show reliable differences, although these comparisons were limited by uneven subgroup sizes and restricted demographic variation. By contrast, attendance frequency showed small but significant differences in technical-expressive perception and emotional perception. More frequent attendees reported higher scores for these two responses, but audience intention did not differ significantly across attendance groups. This pattern suggests that repeated exposure to musical theater is more clearly associated with variation in vocal appraisal and perceived emotional meaning than with stated future intention. A supplementary exploratory analysis further indicated that the positive association between technical-expressive perception and emotional perception became slightly weaker at higher attendance levels. This effect was very small (Δ*R*^2^ = 0.007) and should be interpreted cautiously.

### 5.2 Interpreting the audience response architecture

The proposed Audience Response Architecture clarifies how technical-expressive perception (TEP), emotional perception (EP), and audience intention (AI) are related without reducing them to one general audience response. Emotional perception was more closely related to audience intention than technical-expressive perception was. This suggests that stated intention was not linked most closely to vocal appraisal alone, but to whether the performance was received as emotionally clear and meaningful. This finding refines previous work showing that future attendance intentions are associated with both emotional reaction and performance evaluation, rather than with evaluation alone (Kawase and Obata, 2016). In the context of musical-theater singing, the present results specify this distinction more directly: vocal evaluation remained relevant, but perceived emotional meaning was the response more closely tied to audience intention.

A further implication is that technical-expressive perception should not be treated as a weaker form of audience intention. It captures listeners’ appraisal of vocal delivery, including whether the singing sounds clear, controlled, and expressive. Emotional perception refers to whether the emotional meaning of the performance is received clearly and believably. These two responses were closely related, but they were not interchangeable. Because all variables were measured in a single survey wave, the findings do not show causal order. They describe the observed relations among technical-expressive perception, emotional perception, and audience intention in this sample.

### 5.3 Attendance-related variation in audience response

The attendance-related results add a further layer to the proposed Audience Response Architecture. In the present sample, more frequent attendees reported higher technical-expressive perception and emotional perception, but audience intention did not differ significantly across attendance groups. This indicates that repeated exposure to musical theater was more clearly associated with variation in appraisal and perceived meaning than with stated willingness to return. The pattern is therefore not simply that frequent attendees responded more positively. The differences appeared mainly in vocal appraisal and perceived emotional meaning, rather than in audience intention.

One plausible explanation is that repeated attendance gives listeners more opportunities to develop familiarity with genre-specific vocal and dramatic cues. Research in music perception shows that experience with particular musical genres can shape how listeners perceive timing and follow performance detail. This suggests that long-term exposure may affect how stylistic cues are heard (Danielsen et al., 2022). Repeated exposure has also been shown to increase familiarity, recognition, and liking for unfamiliar musical materials (Mungan et al., 2019). Even short-term exposure can shift evaluations of unfamiliar chords (Smit et al., 2022). In this context, more frequent attendance may help listeners distinguish vocal delivery from its emotional effect more clearly. For less frequent attendees, vocal quality and emotional effect may be experienced as a more combined impression. If so, the higher appraisal and emotional-perception scores among more frequent attendees may reflect greater perceptual familiarity with sung performance, not a stronger general readiness to attend again.

The absence of a parallel shift in audience intention is also consistent with this interpretation. Decisions about attending live performance do not depend only on how clearly a performance is heard or how positively it is evaluated in the moment. They are also shaped by broader motives, including artistic interest and the perceived uniqueness of the live experience (Mulder and Hitters, 2021). Research on concert viewing also suggests that social-presence factors can shape enjoyment beyond the musical material itself (Kulla et al., 2024). This helps explain why more frequent attendees in the present sample showed clearer differences in vocal appraisal and perceived emotional meaning than in audience intention. Their greater familiarity may have helped them separate vocal appraisal from perceived emotional meaning. Stated willingness to return may have remained relatively stable because intention is influenced by a wider set of motives than immediate vocal response alone. Overall, the findings suggest that attendance frequency in the present study was associated more clearly with how performance was appraised and emotionally understood than with stated intention.

### 5.4 Implications

The findings of this study have both theoretical and practical implications. At the theoretical level, the study clarifies how evaluation, perceived emotion, and intention are related in musical-theater listening. It extends work that has linked audience experience to affective and return-related outcomes in music and theater contexts (Chan et al., 2019; Li and Hu, 2025; Tung Au et al., 2017). The present findings add to this work by showing how vocal appraisal, perceived emotional meaning, and stated intention were related in one audience-response model.

At the practical level, the findings concern how listeners perceive musical-theater performance. The results do not indicate that perceived vocal quality is directly linked to audience intention on its own. Rather, listeners appear to respond more strongly when vocal delivery makes the character’s emotion, words, and situation clear. For performance practice, this points to observable features of sung delivery, such as clear diction, controlled dynamics, phrasing that supports the text, and vocal expression that fits the character’s situation (Huang et al., 2024; Moura et al., 2024). This interpretation is consistent with recent research showing that music can communicate more than broad affective tone. It can also convey socially meaningful expressive states, making expressive clarity relevant to audience reception (Pring et al., 2024). For lay listeners, perceived technical quality may therefore be noticed through the emotional effect that sung delivery creates.

### 5.5 Limitations and future directions

Several limitations should be considered when interpreting the findings. The sample was recruited online and was predominantly young, female, and highly educated. Although this profile is relevant to an active segment of the musical-theater audience, it does not represent the wider population of potential audience members. Because some demographic groups were small, the demographic comparisons provide only a limited indication of possible gender, age, and education differences. Future studies with more balanced audience samples would be better placed to examine whether these differences are stable across broader audience groups. In addition, all focal variables were based on self-reported responses collected in a single survey session. This design was appropriate for examining the relations among technical-expressive perception, emotional perception, and audience intention in the present sample, but may also have introduced shared method variance (Cooper et al., 2020; Podsakoff et al., 2024). The study also did not include acoustic measures of singing or ratings from trained vocal experts, so the findings should be interpreted as evidence about perceived vocal delivery rather than measured vocal technique. Because the study was cross-sectional, the findings should not be interpreted as evidence of temporal ordering or causal process. Future longitudinal, repeated-measures, or multi-wave studies would help clarify how these relations develop over time (Caemmerer et al., 2024; S. Zhao, 2024). Audience intention was measured as stated willingness to return or pay rather than as actual attendance or purchase behavior. Future work should incorporate behavioral indicators such as follow-up attendance, ticket purchase, or recommendation behavior (Conner and Norman, 2022). A further limitation concerns the measurement evidence for the proposed three-component structure. Although the retained three-factor solution was interpretable and CFA supported treating the three constructs as distinguishable but interrelated, this evidence should be regarded as supportive rather than definitive. The factor-analytic pattern was theory-informed, while unrestricted extraction suggested a more compact empirical structure. Finally, attendance frequency emerged as a meaningful audience-difference factor, but its explanatory role was limited. The small interaction effect suggests that other individual-difference variables may also shape audience response, including vocal training, familiarity with particular works, and listening goals. Attendance frequency should therefore be treated as an indicator of exposure, rather than as a direct measure of vocal training or perceptual expertise.

Future studies could test the proposed framework in more diverse samples, compare alternative measurement models, and examine how specific vocal features, text, acting, staging, and character situations shape emotional perception and audience intention.

## 6 Conclusion

This study shows that audience response to musical-theater singing involves related but distinct components. Technical-expressive perception, emotional perception, and audience intention were positively related, but they did not play the same role. The findings suggest that stated intention was less closely linked to technical-expressive evaluation alone than to whether the performance was received as emotionally clear, communicatively effective, and meaningful. On this basis, the study proposes an Audience Response Architecture that distinguishes evaluation, perceived emotion, and intention rather than treating them as equivalent audience responses.

The findings also clarify how vocal performance may enter audience experience. In musical theater, singing carries not only sound but also text, character, mood, and dramatic action. The present results suggest that the audience relevance of technical-expressive quality becomes clearer when it helps communicate emotional and dramatic meaning. The attendance-related findings add a further qualification. More frequent attendees reported higher technical-expressive perception and emotional perception, but not higher audience intention. This suggests that repeated exposure may be more closely associated with appraisal and perceived meaning than with stated willingness to return. Overall, the study points to an audience-oriented implication for musical-theater practice: technical control appears especially relevant to audience experience when it helps listeners understand the character’s emotion, words, and situation.

## Data Availability

The datasets presented in this study can be found in online repositories. The names of the repository/repositories and accession number(s) can be found below: https://osf.io/23f4m/overview?view_only=b7f9534ba3e54052a34e6706cd2877ed.
