# Supplementary material for: Evaluative and emotional routes to audience intention in musical-theater singing: An integrated response architecture
Source: Front Psychol. 2026 Aug 4;17:1870336. doi: 10.3389/fpsyg.2026.1870336 (PMC13481799; doi:10.3389/fpsyg.2026.1870336)
Supplement: Supplementary file 1 [file Supplementary_file_1.docx]

**SI file. English version of the audience questionnaire**

Thank you for agreeing to participate in this research project. This questionnaire examines how audiences perceive different singing styles in musical theatre, especially in relation to expressive delivery, emotional communication, and audience acceptance. The questionnaire was developed for this study and was administered in Mandarin Chinese; the present document provides the English version for reference. Participation is voluntary. You may stop at any time without penalty. No direct personal benefit is offered. All responses are confidential and will be used only for academic research. Please answer the questions according to your own genuine perceptions and experiences. The questionnaire takes approximately 5–8 minutes to complete. (The original questionnaire included an age-screening option for respondents under 18, but only adult responses were retained for analysis.)

Section A. Demographic information

Q1. What is your gender?

A. Male

B. Female

Q2. What is your age?

A. Under 18

B. 18–30

C. 31–45

D. 46 and above

Q3. What is your educational background?

A. High school or below

B. Junior college / Bachelor’s degree

C. Master’s degree or above

Q4. Approximately how often do you watch musical theatre each year?

A. Less than once

B. 1–3 times

C. 4–6 times

D. 7 times or more

Section B. Technical–expressive perception (TEP core items)

Q5. How important do you think singing technique is for character portrayal in musical-theatre performance?

A. Very important

B. Quite important

C. Moderate

D. Slightly important

E. Not important at all

Q6. Do you think the use of pop-derived vocal techniques, such as breathy tone or mixed voice, helps enhance the artistic expressiveness of musical-theatre performance?

A. Very helpful

B. Quite helpful

C. Moderate

D. Slightly helpful

E. Not helpful at all

Q7. How do you evaluate the influence of musical-theatre singing technique on the clarity of emotional expression in stage performance?

A. Very clear

B. Quite clear

C. Moderate

D. Not very clear

E. Not clear at all

Section C. Emotional perception (EP core items)

Q8. Do you think vibrato rate and timbral changes in musical-theatre performance can accurately convey a character’s emotion?

A. Completely

B. Mostly

C. Moderately

D. Rarely

E. Not at all

Q9. When watching a musical, can you perceive changes in the character’s emotion through the actor’s singing?

A. Completely

B. Mostly

C. Moderately

D. Rarely

E. Not at all

Q10. Does the use of pop singing techniques in musical theatre make it easier for you to feel emotional resonance with the performance?

A. Very easy

B. Quite easy

C. Moderate

D. Quite difficult

E. Very difficult

Section D. Audience intention (AI core items)

Q12. Would you prefer to watch musicals that incorporate pop singing styles?

A. Very much

B. Quite a lot

C. Moderate

D. Not very much

E. Not at all

Q15. If a musical incorporated more pop elements in its singing style, would you be willing to buy a ticket to watch it?

A. Very willing

B. Quite willing

C. Moderate

D. Not very willing

E. Not willing at all

Section E. Additional descriptive items

Q11. In terms of emotional expression, which vocal approach do you think is more commonly used in musical-theatre performance?

A. Pop vocal techniques (e.g. falsetto, light mix)

B. Traditional musical-theatre techniques (e.g. chest voice, head voice)

C. A combination of both

Q13. Does innovation in singing style affect your acceptance of musical-theatre ticket prices?

A. Very much

B. Quite a lot

C. Moderate

D. Not very much

E. Not at all

Q14. Do you think the use of pop singing styles in musical theatre can attract more young audiences?

A. Definitely yes

B. Probably yes

C. Uncertain

D. Probably not

E. Definitely not
